# Effect of social media marketing on online travel purchase behavior post-COVID-19: mediating role of brand trust and brand loyalty

**DOI:** 10.1186/s43093-023-00192-6

**Published:** 2023-03-28

**Authors:** Mohd Azhar, Rehan Husain, Sheeba Hamid, Mohd Nayyer Rahman

**Affiliations:** 1 Department of Commerce, Aligarh Muslim University, Aligarh, 202002 India; 2 Jaipuria Institute of Management, 226010 Lucknow, India

**Keywords:** Social media marketing, Brand trust, Brand loyalty, Purchase intention, COVID-19

## Abstract

The present study intends to unleash the influence of social media marketing (SMM) on purchase intention (PI), brand trust (BT) and brand loyalty (BL) in the setting of online travel booking websites. It also analyses the mediating effect of BT and BL in the relationship between SMM and PI. This study also examines the importance of trust and loyalty in the suggested model, which adds to the current research in this area. A self-administered questionnaire was employed to collect the data from the users of online travel booking websites, and the study rested upon 397 valid responses. Data were analyzed using partial least squares structural equation modeling (PLS-SEM) through Smart PLS v.3.2.6. The findings reveal that SMM has a favorable and substantial impact on BT and BL, influencing COVID-19 purchase intention. As per the findings, BT and BL's beneficial influence on the purchase intention of arranging travel on social media was discovered. Moreover, it was also confirmed from the results that BT and BL mediate the relationship between SMM and PI. Therefore, SMM significantly impacts online trip booking purchase intentions with increased BT and BL levels. Finally, several theoretical and managerial implications can be delineated from the findings of this study for industry and academia.

## Introduction

The exponential growth of information and communication technology (ICT) has resulted in substantial shifts in the day-to-day operations of firms in the tourism and hospitality sector. These shifts have been chiefly positive [122], subsequently, the overall experience of tourists has also exalted [20, 107]. ICT has been instrumental in increasing the sales of tourism-related products and services [31, 98]. It is anticipated that "72% of the entire sales of tourism products will be done online by the year 2025, and the revenue will increase at a pace of 13.9% annually (CAGR 2021–2025), which will result in the projected market volume of US$ 909,235 million by the year 2025" [105]. Due to the rampant advancement in ICT-enabled applications, many easily accessible platforms have evolved abruptly, viz., ticket booking sites, online tour operating and shopping websites, product review websites, chat forums, blogs, and other social media sites [24]. Because of the unique participatory nature of social media, online consumers have had unparalleled access to information about products, companies, and services in the past several years [36]. Customers increasingly turn to social media to find the helpful information before purchasing [94]. They incorporate this information into brand evaluations and purchase decisions [104]. The popularity of social media has skyrocketed in recent years [18, 110], and during COVID-19, a surge in social networking sites has been recorded [63, 75]. Therefore, it has become crucial to study the effects of social media in the present time.

It is generally acknowledged that the tourism sector is not only a significant contributor to economic expansion but also a key generator of wealth, means of subsistence, and income [18, 77]. Over the course of the last several years, it has seen remarkable expansion and diversification, becoming one of the economic subfields with the highest rate of expansion worldwide [91]. However, the sudden onset of the COVID-19 pandemic had a profoundly negative impact on both the state of the global economy [115, 116] and the environment on a worldwide scale [113]. As a direct consequence of this, the expansion of travel and tourism-related activities was stymied and eventually came to a complete standstill [12, 48]. The constraints imposed by COVID-19 had a devastating effect on the expansion and growth of the world economy [114−116]. The COVID-19 pandemic is estimated to have caused the loss of almost 47.7 million tourism-related employment in South Asia, as stated in a report published by the World Bank. In addition to this, the report states that the Indian economy saw a potential loss of 43.4 billion US dollars in GDP [118]. It is anticipated that it will take at least two years to return to the levels that existed before the pandemic [106]. Considering the negative consequences of COVID-19 on travel and tourism industry, it becomes quite relevant to study this industry in the post-COVID-19 phase and how online travel purchase behavior contributes to the revival of the travel industry. Hence, the present study sincerely meets this emerging need by unleashing the influence of SMM on PI, BT and BL in the setting of online travel booking websites.

The influence of social media on consumer behavior is all-pervasive [11]. Therefore, its usage is becoming more prevalent in the branding and marketing industry [104] to earn a positive brand image, brand trust, and brand loyalty [90]. To reach a wider audience, companies increasingly use Facebook, Instagram, and Twitter to develop brand fan pages. It has spawned a new category of marketing strategies known as "social media marketing" (SMM) [57]. Since social media is used by more than half of the world's population, the travel and tourism industry has much scope to grow in this area and utilize it as a part of its marketing strategy [57]. Although some studies have examined the branding and brand equity benefits of SMM [22, 67, 126], few researchers have described how and why SMM affects purchase behavior, brand loyalty, and brand trust [32, 56, 62, 81, 117]. The present study is different from previous studies in many ways. First, it assesses the effect of SMM on purchase behavior, brand trust, and brand loyalty in the travel and tourism industry. Second, it examines the role of brand trust and brand loyalty as a mediator between SMM and purchase behavior. Third, post-COVID-19 travel purchase behavior has been assessed, unlike the previous studies.

## Theoretical background

The act of making purchases of products and services via the use of the internet is referred to as "online shopping behavior" [108]. It pertains to the psychological state of the online purchaser [72]. People all around the globe have been convinced to make subtle adjustments to their behaviors and attitudes as a result of social media [78]. The purchase behavior of consumers around the world is changing [42], and an increasing number of people are making their purchases of goods and services online [34]. As a result of the COVID-19 pandemic, customers have been encouraged to make more purchases on online marketplaces [42]. This has caused an explosion in online purchases. This holds true for the travel sector as well. Customers tend to have positive intentions regarding using the internet for travel searches and booking [99]. Social media has substantially facilitated consumers' ability to search for and purchase travel products [112]. Social media enhances information interchange, decreases uncertainty related to travel purchases, and radically alters individual travel purchase behavior [52, 124].

Several research has been conducted in the Western world to understand online travel purchase behavior [60, 66, 95, 96] while emerging economies like India left unstudied in this regard. Khare et al. [64] and Sadiq et al. [99] made sincere attempts, but the context of their studies was different from the present one, and they did not provide a comprehensive picture of online travel purchase behavior. To date, no study has been carried out in the Indian context that has measured the influence of SMM on online travel purchase behavior post-COVID-19, taking BT and BL as mediating variables. Hence, the present study responds to this gap with the intention of broadening the scope of the existing stock of knowledge on the subject concern. The researchers posit that knowing consumers' online travel purchase behavior will provide a deeper insight into their tastes and preferences and lessen the complexities of online shopping activities for travel products.

## Hypotheses development

### Social media marketing and purchase intention

Consumers' readiness to buy a product is referred to as purchase intention (PI) [6]. To put it another way, the likelihood of a customer making a purchase is termed purchase intention. Consumers' propensity to buy travel products and services over social media is considered in the present research. As Ajzen [6] noted, consumers' motivation plays a key role in shaping PI, which in turn influences behavior, for example, the higher the PI, the more positive the action [99]. It is extensively shown in the literature that PI may accurately predict real online purchase behavior [7, 16, 17].

In addition, the study that has been conducted on the topic of purchasing trips online has identified PI as one of the indicators of actual behavior. This recognition comes from the fact that PI is a predictive index [4]. In addition, Ajzen [6] claimed that consumers with greater PI are more likely to do the desired action, consequently, it is thought that customers with more powerful PI reflect real behavior [120]. Due to difficulties in evaluating actual consumer behavior, Agag and El-Masry [3] recommended utilizing PI since PI is one of the most important predictors of real behavior. Therefore, in the present study, purchase intention has been used to measure purchase behavior.

SMM can be characterized as having a "considerable role in building trust and the consumer-brand relationship, which lead to positive business outcomes in turn" [26]. Studies on SMM investigated numerous behavioral consequences, including individual positive behavior [25, 67, 126]. Many previous studies have found a significant association between SMM and behavioral intention (purchase intention) [25, 26, 103]. Therefore, based on the literature, it is hypothesized that:

#### H_1_

Social media marketing positively influences purchase intention.

### Social media marketing and brand trust

Through the use of SMM, customers are transformed into marketers and promoters. They create, manage, and distribute online information related to companies, products, and services [56, 58]. A number of past studies investigated the association between SMM and trust in different industries other than travel and tourism [32, 81, 121]. The present study has tried to establish a link between SMM and brand trust in online travel purchase behavior. Trust is one of the most important factors in making sales [65]. It is consistent with SMM's intended purpose of facilitating enhanced lines of communication among businesses' marketing divisions to forge lasting bonds with their clientele [32, 41]. Therefore, based on the literature, it is hypothesized that:

#### H_2_

Social media marketing positively influences brand trust.

### Social media marketing and brand loyalty

Brand loyalty is "an attachment to a particular company and its products" [68]. According to Oliver [85], brand loyalty is "a deeply held commitment to re-buy or patronize preferred product/services consistently in the future". Brand loyalty is the willingness to repurchase a product and show continued interest in it [33]. Loyal customers talk favorably about the brand over social media [123]. A direct association between SMM and brand loyalty has been shown in the marketing literature [57]. Ebrahim [32] found a significant and direct association between SMM and brand loyalty. Customer relationship quality and behavior outcomes were shown to be favorably impacted by SMM and customer experience, as reported by Wibowo et al. [117]. Brands and consumers that can communicate effectively with one another or engage in more two-way interaction with one another regarding goods and services have a compelling perspective connection from a brand-building perspective [53]. Therefore, the better the contact and relationship between the brand and the customer, the more devoted the consumer will be to the brand [57]. Therefore, based on the literature, it is hypothesized that:

#### H_3_

Social media marketing positively influences brand loyalty.

### Brand trust and purchase intention

The term "brand trust" refers to a customer's implicit faith that an online service provider follows through on their expectations about the quality of the service they get in exchange for their payment [3]. Furthermore, it is described as a process that evolves and is informed by prior exposure to a product or service [9]. One of the most critical aspects of studying consumer behavior while purchasing goods online is brand trust [23]. Previous studies on online travel purchases have shown a favorable correlation between PI and trust [2, 3, 13]. Therefore, based on the literature, it is hypothesized that:

#### H_4_

Brand trust positively influences purchase intention.

### Brand loyalty and purchase intention

Brand loyalty is a consequence of the emotional connection between a customer and a brand [79]. According to Li and Green [71], in the face of fierce competition, an organization's ability to maintain customer loyalty is crucial. As pointed out by Saili et al. [100], loyalty might be seen as "flying on two wings: (behavioral and attitudinal)". Attitudinal loyalty is the propensity to favor one brand above another, as shown by the likelihood that the preferred brand will be purchased [84]. However, Rundle-Thiele and Mackay [97] argue that customers who are devoted to a brand will also show loyalty throughout the purchasing process. According to what Nam et al. [82] have indicated, brand loyalty is a behavioral structure related to intentions toward recurrent purchases. Customers that are committed to a brand are inclined to purchase it again. Therefore, based on the literature, it is hypothesized that:

#### H_5_

Brand loyalty positively influences purchase intention.

### Brand trust and brand loyalty

Brand trust is defined as "the willingness of the average consumer to rely on the ability of the brand to perform its stated function" [21]. In their study, Chaudhuri and Holbrook [21] found that a high degree of brand loyalty was highly associated with clients who exhibited positive and confident emotions. This way, consumers may feel comfortable committing to and identifying with a brand [57]. In the words of Gunelius [43], "building relationships on the social web is practically a guaranteed way of deepening brand loyalty". Trust in a brand ultimately results in brand loyalty or commitment [74] because trust fosters the development of highly valued trade relationships [80]. In this way, SMM boosts brand trust, influencing brand loyalty. Therefore, based on the literature, it is hypothesized that:

#### H_6_

Brand trust positively influences brand loyalty.

### Mediating role of brand trust

The tourism industry employs several sorts of marketing and advertising to influence consumers' decisions to travel [14, 54]. To better understand the impact of mediators on purchase intention in the context of the tourism sector, this research expands on the proposed framework within the context of COVID-19. Social media, when viewed through the lens of the tourism sector, provides an opportunity for marketers to dwell on this. Using SMM, businesses can have a two-way conversation with their target audience, which fosters trust and loyalty [87]. SMM entails complementary processes that provide robust engagement and connection between customers and businesses [47]. Satisfaction, commitment, and trust mediate behavior and outcome in relationship marketing [86]. Lin and Lu [73] hypothesized that trust has a significant impact on repurchase intention. Chiu et al. [27] discovered that different levels of customer trust might influence a consumer's propensity to repurchase or revisit a brand. Researchers have found that brand trust in brands may affect both their perceptions and their actions [1]. Since brand trust is an emotional state that develops during the provision of service [19], several writers have proposed it as a mediator between two (or more) variables [1]. SMM can have an indirect impact on purchase intention via the mediating effect of brand trust. Therefore, based on the literature, it is hypothesized that:

#### H_7_

Brand trust mediates the relationship between social media marketing and purchase intention.

### Mediating role of brand loyalty

SMM is an interactive tool that helps cultivate customer connections and brand loyalty in the context of a social media-based brand community [58]. The close relationship between brands and their consumers on social media has increased brand loyalty [57]. By analyzing summary measures of brand purchase patterns, striking regularities in customers' preferences for certain brands across a wide range of items were discovered [28]. Jacoby and Kyner [59] defined brand loyalty as a subset of repeat purchases and a multidimensional entity comprising attitude components. Consumers devoted to a specific brand are driven to buy from the company repeatedly [28]. It has been found by Cheung et al. [25] that SMM has a significant role in fostering customer-brand engagement, repurchase intention, and continued searches among smartphone companies in China and Hong Kong. According to the findings of a few tourism-related research, a loyal client base leads to an increased likelihood of future visits [8, 56]. Brand loyalty was found to be a mediating factor between social media marketing and tourism-related purchase intention in an investigation by Abou-Shouk and Soliman [1]. Hence, in the present study, a mediating effect of brand loyalty on purchase intention has been conceptualized. Therefore, based on the literature, it is hypothesized that:

#### H_8_

Brand loyalty mediates the relationship between social media marketing and purchase intention.

**Fig. 1:**
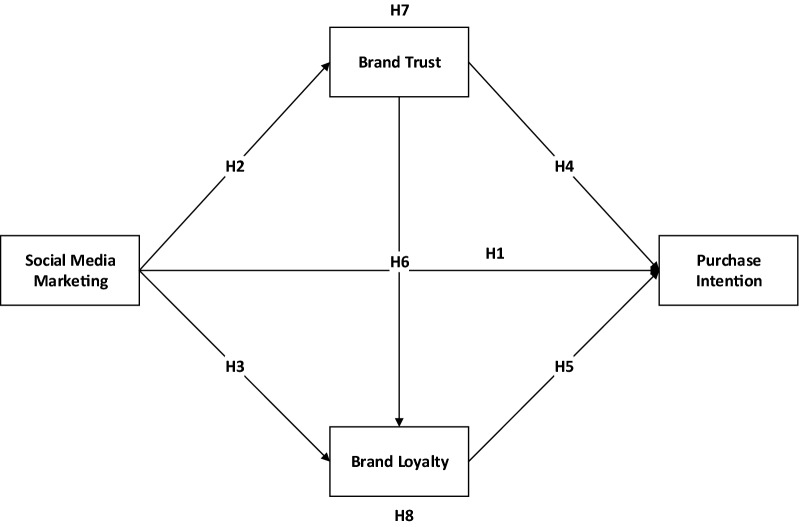
Theoretical framework *Source*: The authors

The hypotheses can be presented as shown in Fig. 1.

## Research methodology

The primary data for empirical analysis was collected via a self-administered survey questionnaire, which was conducted to evaluate research hypotheses created within a given theoretical framework.

### Survey instrument

The social media marketing scale was adopted from the studies of Seo and Park [102], Kim and Ko [65] and contain four items. The brand trust scale was taken from Laroche et al. [69], Chaudhuri and Holbrook [21] and has four items. The brand loyalty scale employed in the current study was taken from Ailawadi et al. [5] with four items. Lastly, the purchase intention scale was taken from Venkatesh et al. [111], Taylor and Todd [109] which contains four items. The language and wording of the adopted items were modified to match the suitability of the present study. All the scales adopted were validated and empirically tested. Hence, using them in the Indian context requires additional validity tests, which researchers have performed with great caution. All the items were collected on a seven-point Likert scale ranging from strongly disagree (1) to strongly agree (7). It is mentioned in several studies that, instead of using a five-point Likert scale, it is a good practice to use a seven-point Likert scale. When the constructs are subjective, a seven-point scale measures the items in a more sophisticated manner [10, 45, 76].

### Research panel

The authors focused on the consumers of the top five most prominent travel websites in India, as demonstrated by several industry publications, to guarantee that the present study would apply to the Indian market [30, 40]. This method also allowed for more precise participant identification, narrowing down the consumers who had previously booked on the selected top five traveling websites [37, 88]. IRCTC, Yatra online, MMT, Cleartrip, and Expedia were all targets. This strategy targeted various consumers across age groups and gender. The Indian tourism industry is also of practical importance, as Indian tourism has grown significantly high in recent years [55]. India, the developing economy, was chosen for the present study because of projections that by 2027 it would have the world's fourth-largest GDP-based tourism economy after China, the USA, and Germany [119]. India's healthcare system, economy, labor market, and tourism and hospitality sectors have all been impacted by the COVID-19 pandemic. For instance, as of the end of September 2020 in India, out of almost 6 million confirmed cases, about 940,000 were considered active, over 5 million had recovered, and 97,529 had died [83]. Second, the increase in COVID-19 cases has caused the IMF to increase the rate at which it predicts India's GDP to fall in 2020–21 from 4.5% to 10.3%. Lastly, based on several reports, white papers, and published systematic reviews, it is widely accepted that tourism is one of the best sectors to see exponential growth.

### Data collection and research design

To test the hypotheses of the proposed model, the authors employed a questionnaire in this investigation. First, only those respondents who said they had used social media to look for travel information were included in the survey. Secondly, it was required that they have visited key tourist websites in India (e.g., MMT, Cleartrip, etc.). Pilot research, including 57 participants in 13 days of data collection, was conducted before the main study. It was determined that there were several issues with some of the statements. Hence, they were either removed based on the factor loadings or the language was modified. Thus, it solved the problems with the clarity or readability of the questionnaire's items.

Data for the empirical examination was collected from February 2022 to April 2022 by self-administered questionnaires from a convenience sample [93]. A 59.23% response rate was achieved with 397 valid replies from 670 questionnaires. The researchers got the maximum responses from the 31–40 age group. It was clear that male respondents were 65% while female respondents were 35% in number. See Table 1 for more detail.

**Table 1:** Demographic profile *Source*: Primary data

Variable description	Frequency	Percentage (%)
Gender		
Male	258	65
Female	139	35
Age group		
20–30 years	98	24.68
31–40 years	122	30.73
41–50 years	101	25.44
> 60 years	76	19.14
Platform used for travel booking		
MMT	113	28.46
Cleartrip	77	19.39
Yatra online	48	12.09
Expedia	69	17.38
IRCTC	90	22.67
How long have you been using social media for travel booking?		
1–5 years	115	29
5–10 years	226	57
> 10 years	56	14

### Non-response bias

This research examines non-response bias by comparing the demographic features of early and late respondents (age, gender, and measurement items), as proposed by Armstrong and Overton [15]. There are no statistically significant differences (*p* > 0.05) between the characteristics of early and late participants, as determined by a Chi-square test. Also, *t*-tests showed no statistically significant differences (*p* > 0.05) between early and late responders on any measurement items. Hence, it can be said that this study is free from non-response bias.

### Common method bias

Next, the authors used both criteria to assess how well common method variance (CMV) performed. The authors started by using Harman's single-factor approach. The total variation explained by a single component was 34.6% (below 50%); according to the findings, it can be said that CMV is not an issue in this study [89]. As a second step, CMV was evaluated using the variance inflation factors (VIFs). The VIF values in this study were below 5, as shown in Table 2, confirming the absence of CMV and/or multi-collinearity [46, 101].

**Table 2:** Results of measurement model: reliability and validity *Source*: Primary data

Construct used	Measurement items	Factor loadings	AVE	CR	Cronbach's alpha	VIF
Social media marketing	SMM1	0.893***	0.778	0.861	0.965	1.32
	SMM2	0.869***				
	SMM3	0.914***				
	SMM4	0.916***				
Brand trust	BT1	0.907***	0.762	0.853	0.845	1.65
	BT2	0.923***				
	BT3	0.930***				
	BT4	0.789***				
Brand loyalty	BL1	0.902***	0.765	0.813	0.963	2.43
	BL2	0.896***				
	BL3	0.876***				
	BL4	0.868***				
Purchase intention	PI1	0.941***	0.739	0.943	0.860	2.67
	PI2	0.960***				
	PI3	0.935***				
	PI4	0.845***				

Significant at ***p < 0.01

## Results

Partial least squares structural equation modeling (PLS-SEM) was used to evaluate the study model, and Smart PLS v.3.2.6 was used to analyze the data. The smart PLS is more advanced in handling complicated models, non-normal data distributions, exploratory and predictive studies with small samples, and so on [46]. Higher statistical power [92] and the capacity to test hypothesized relations [101] are only two of the reasons why PLS-based analysis has been widely adopted by previous (tourism) scholars [46, 92].

### Measurement model analysis

Reliability and validity are the most important criteria that are checked before further analysis in smart PLS. Hence, based on the previous scholars and their published studies, the authors have reviewed the threshold value and matched the value obtained in the current study. The results are shown in Tables 2 and 3. The factor loading of each construct was over the critical value of 0.70 to be considered significant [46]. When AVEs values for a given concept were over the threshold value of 0.50, indicating acceptable convergent validity [38, 46, 93]. Hence, it is concluded that the constructs in question were really valid. Scale reliability was high, with Cronbach's alpha values ranging from 0.845 to 0.965 and composite reliability values ranging from 0.813 to 0.943 (Table 2).

**Table 3:** Results of measurement model: discriminant validity-HTMT criteria *Source*: Primary Data

Factor	SMM	BT	BL	PI
SMM	**0.856**			
BT	0.759	**0.802**		
BL	0.725	0.795	**0.786**	
PI	0.621	0.726	0.636	**0.712**

Bold values are HTMT ratios

Fornell and Larcker's [38] criteria for evaluating discriminant validity was used initially, and then the Heterotrait-Monotrait ratio of correlations (HTMT) was used [46]. Table 3 shows that the square roots of the AVEs for each construct are higher than their corresponding intercorrelations. The HTMT readings were also lower than the cut-off value of 0.9, demonstrating discriminant validity.

### Structural model evaluation

Several criteria were used to assess the structural/path model. Standardized root means square residual (SRMR) was supported as the approximation model fit criteria by Henseler et al. [50]. In their work, the authors proposed that an SRMR of 0.10 indicates a satisfactory model fit. The present study's findings show a satisfactory model fit (SRMR = 0.081), thus supporting this interpretation. Additionally, R2 and Q2 values of predicted variables were used to evaluate the model's predictive ability. All of the R2 values exceeded the threshold of 0.10 that was proposed by Falk and Miller [35] (SMM: 0.42, BT: 0.69; BL: 0.67; PI: 0.54). Positive Stone-Geisser Q2 values for all endogenous constructs (SMM: 0.14, BT: 0.17, BL: 0.16; PI: 0.19) further support that the model has a strong predictive ability [46].

The path analysis of the proposed model is presented in Table 4. Consistent with H_1_, social media significantly influences purchase intention (*β* = 0.455). All the proposed hypotheses were supported in our case except H_6_, which shows the relationship between brand loyalty and brand trust.

**Table 4:** Results of PLS-SEM *Source*: Primary data

No	Hypothetical relationships	Estimates (*β*)	*t*-value	*f*^2^	Remarks
H_1_	PI < – SMM	0.455***	8.34	0.34	Supported
H_2_	BT < – SMM	0.178***	6.28	0.28	Supported
H_3_	BL < – SMM	0.367***	7.31	0.31	Supported
H_4_	PI < – BT	0.189***	5.34	0.34	Supported
H_5_	PI < – BL	0.267***	9.29	0.29	Supported
H_6_	BL < – BT	0.231***	8.33	0.33	Not supported

Significant at *** p < *0.01*

Next, it was checked how much influence exogenous variables have on a dependent variable (f2), classifying the strength of that influence using Cohen's thresholds of 0.02 (weak), 0.15 (moderate), and 0.35 (strong) [46]. The f2 values for the impacts of the pathways were between 0.28 and 0.34, showing significant relationships. For detailed information, see Table 4.

### Mediation analysis

Mediation was evaluated using a product-of-coefficients method implemented in PROCESS (e.g., [49] with a confidence interval of 95% based on 5000 bootstrap samples confidence interval (CI). The absence of a null CI showed support for H_7_ and H_8_ via mediation for the indirect impact. From Table 5, it can be seen that both hypotheses were supported in the case of mediation effect testing. Brand trust was found to be more assertive in the case of the correlation between social media marketing and purchase intention (*β* = 0.61), followed by the relationship between social media marketing and purchase intention mediated by brand loyalty (*β* = 0.45).

**Table 5:** Mediation model analysis

No.	IV	Mediator	DV	*β*	S.E	LLCI	ULCI	Remarks
H_7_	SMM	BL	PI	0.45	0.059	0.132	0.341	Supported
H_8_	SMM	BT	PI	0.61	0.063	0.235	0.432	Supported

## Discussion and conclusion

The present study intended to unwrap the influence of SMM on PI in the context of online travel booking websites post-COVID-19. In addition, it also analyzed the mediating effect of BT and BL in the relationship between SMM and PI. Although some previous studies have examined the impact of SM on loyalty, satisfaction, purchase decision, purchase intention and purchase behavior [39, 44, 51, 125], the variables used in those studies were different, and they were carried out in different settings. Some studies talked about online travel purchase behavior, but they were carried out in the Western world [60, 66, 95, 96]. To the best of researchers' knowledge, no study has been carried out in the Indian context that has analyzed the influence of SMM on online travel purchase behavior that too in the post-COVID-19 and has also measured the mediating effect of BT and BL in the relationship between SMM and PI. For the first time, an empirical investigation has been conducted into how brand trust and brand loyalty to a brand affect purchase intention in the Indian travel industry after COVID-19.

The findings of the study unveil that out of the eight hypotheses, seven hypotheses (H_1_, H_2_, H_3_, H_4_, H_5_, H_7_, H_8_) support the evidence, while one hypothesis (H_6_) does not support it. Hypotheses H_1_-H_6_ were related to the direct effect, while hypotheses H_7_ and H_8_ were related to the indirect effect (mediating effect). Results show that the direct effect of SMM on PI is the highest (*β* = 0.455, *t*-value = 8.34, *p* < 0.01), confirming H_1._ Thus, SMM is the most influencial and strongest predictor of PI. This outcome is aligned with the previous studies [25, 26, 70, 92, 103]. SMM also has a significant and positive impact on BL (*β* = 0.367, *t*-value = 7.31, *p* < 0.01), confirming H_3_. This outcome confirms the studies of Madeline et al*.* (2019) and Kazmi and Khalique [61], which theorized the function of social media to build engaged and loyal customers. The effect of SMM on BT also shows a significant and positive association (*β* = 0.178, *t*-value = 6.28, *p* < 0.01), confirming H_2_. This outcome is in accordance with previous studies [32, 41, 65]. Customers have historically had a hard time putting their faith in brands on social media, but as these networks have evolved and attracted more members, businesses have worked to make them more trustworthy. Having faith in a company's brand may be a key factor in establishing a solid foundation for a long-term engagement with the business.

BT has a significant and direct effect on PI (*β* = 0.189, *t*-value = 5.34, *p* < 0.01), confirming H_4_. This outcome is in accordance with previous studies [2, 3, 13]. Similarly, BL also has a significant and direct effect on PI (*β* = 0.267, *t*-value = 9.29, *p* < 0.01), confirming H_5_. This outcome is aligned with the study of Ibrahim et al. [57]. While the direct association between BT and BL came out insignificant, thus rejecting H_6_. Moreover, the results of the present study also reveal that social media's indirect effect on purchase intention is mediated through BT. This finding is align with the studies of Le et al. (2021) and Coelho et al. [29]. Similar is the case for the BL. Thus, confirming H_7_ and H_8_.

It is evident that social media marketing has become part and parcel of our daily life. Hence it is the perfect time that marketing managers must incorporate them in planning their marketing and advertising endeavors. The travel and tourism industry is one of the fastest-growing industries worldwide; hence it should take full leverage of social media marketing. Several studies conducted in the past have already established the fact that brand trust and brand loyalty are considered as most engaging and impactful parameters that need to be focused upon. Therefore, it would be beneficial to use the collaborative combination of trust and loyalty in managing and luring consumers toward the targeted websites.

### Implications

#### Theoretical implications

The present study provides substantial theoretical support and adds to the body of existing stock of literature on SMM, BT, and BL by developing a theoretical model incorporating the mediating role of loyalty and trust in purchase intention. Academicians and researchers may benefit greatly from the theoretical framework established in the present study since it uncovers the influence of SMM on PI after COVID-19. What this research discovered sheds great insight into the relevance of social media-based involvement during pandemics, particularly concerning the development of content in collaboration and the likelihood of purchase. In addition, this study contributes to the knowledge of the mediating functions of trust and loyalty during pandemics. Many previous studies have shown that social media has a direct and positive influence on engagement, which leads to the establishment of brand loyalty, but empirical insight into the relationship of suggested model concepts is limited. Moreover, this study expands upon the pre-existing framework and offers crucial recommendations for tourism researchers who wish to include the mediators. The present study advances existing theory by incorporating social media, tourists' participation with purchase intentions, brand trust, and brand loyalty after the COVID-19 crisis. Developing the tourism industry's economic resilience in the face of a pandemic might benefit from more investigation of the interconnections between the abovementioned factors.

#### Managerial implications

Practitioners in tourism (marketing) might also get helpful information from this study, particularly in times of crisis or pandemics. First, this research highlights the significance of social media in the travel and tourism industry's contribution to its growth. The findings prove that social media is a worthwhile investment for businesses. Thus, it is recommended that tourism marketers create various marketing strategies and approaches that highlight the importance of brand loyalty in tourism via social media during a crisis or a pandemic like COVID-19. Second, the research demonstrates the importance of brand trust in fostering credibility and purchase intention, proving strategic value in fostering customer/brand connections throughout pandemics. Managers are urged, for instance, to keep and develop several online platforms, such as online brand communities and so on. This study recommends that managers keep targeting those visitors who started using new brand-linked platforms during the pandemic and want to use them again after the crisis has passed by using the same channels. These findings inform businesses about the efficacy of SMM tactics in strengthening relationships between brands and potential travelers during times of crisis. The results support the involvement of BL and BT as mediators. It is thus essential for marketers and tourism managers to think about how their marketing practices/strategies and advertising may be strengthened during pandemics to improve BT and boost businesses. The travel sector must use new technologies, such as the implementation of touchless operations at all available contact points in the booking process. As a result, the use of social media by tourist marketers is essential.

The results of the research make it abundantly clear that the use of social media platforms has evolved into an integral component of the process of sharing information and experiences linked with the tourism industry. As a result, businesses and service providers in the travel and tourism industry need to have a significant presence on social media in order to better understand their consumer base, meet the evolving requirements of their customers, and cultivate close relationships with prospective clients. If they did so, they would have good word of mouth and would be able to obtain a competitive edge over their rivals.

Therefore, marketing managers of the websites should pay great attention to this particular area of emphasis. In this context, businesses that provide services linked to travel and tourism need to place a strong emphasis on collecting timely feedback about their products and services in order to raise the bar for the quality of such offerings. Additionally the role of BT can be enhanced in the present scenario. Once the BT is established the band loyalty can be garnered from it. Those that work on the websites for social media platforms might also get benefits from the conclusions of this research. To assist users, managers might use social networks like communication tactics or brand communities. In the event of a pandemic, it encourages brand awareness and increases the likelihood of repeat business and referrals to booking websites.

### Limitations and future research directions

Several caveats exist with this study that must be addressed by future researchers. First, the present study is cross-sectional, which can limit the findings. A customer survey was conducted after lessening the coronavirus preventative measures in India, such as travel restrictions, isolation, and quarantine. Accordingly, following a pandemic, more generalizable conclusions can be obtained through longitudinal studies. Second, the moderating effect of any factor was not investigated in this study. Researchers in the future should include moderating factors such as gender, age, socio-economic status, and culture. Third, additional elements like brand love and satisfaction might be investigated as mediators in future research. Fourth, other antecedents of brand equity could be explored in future studies to shed light on the topic, such as brand prominence, brand association, and brand awareness. Fifth, this study only recruited participants from India; thus, any extrapolations should be made with caution. Therefore, it is recommended that the investigation be repeated in different countries with varying cultural settings. Finally, it is worth noting that COVID-19 is not without its predecessors. Nonetheless, the tourist sector may emerge from the ashes of COVID-19, looking quite different from its pre-outbreak incarnation; therefore, it will be vital to revisit this issue in the future.

## Data Availability

The authors declare that all types of data used in this study is available for any clarification. The authors of this manuscript are ready for any justification regarding the data set. To make available of the data set used in this study, the seeker must mail to the mentioned email address. The profile of the respondents is completely confidential.
